# Targeted Therapy of Uveal Melanoma: Recent Failures and New Perspectives

**DOI:** 10.3390/cancers11060846

**Published:** 2019-06-18

**Authors:** Michela Croce, Silvano Ferrini, Ulrich Pfeffer, Rosaria Gangemi

**Affiliations:** IRCCS Ospedale Policlinico San Martino, 16132 Genoa, Italy; michela.croce@hsanmartino.it (M.C.); silvanodomenico.ferrini@hsanmartino.it (S.F.); patologia.molecolare.integrata@gmail.com (U.P.)

**Keywords:** uveal melanoma, driver mutations, signaling pathways, targeted therapies

## Abstract

Among Uveal Melanoma (UM) driver mutations, those involving *GNAQ* or *GNA11* genes are the most frequent, while a minor fraction of tumors bears mutations in the *PLCB4* or *CYSLTR2* genes. Direct inhibition of constitutively active oncoproteins deriving from these mutations is still in its infancy in UM, whereas *BRAFV600E*-targeted therapy has obtained relevant results in cutaneous melanoma. However, UM driver mutations converge on common downstream signaling pathways such as PKC/MAPK, PI3K/AKT, and YAP/TAZ, which are presently considered as actionable targets. In addition, *BAP1* loss, which characterizes UM metastatic progression, affects chromatin structure via histone H2A deubiquitylation that may be counteracted by histone deacetylase inhibitors. Encouraging results of preclinical studies targeting signaling molecules such as MAPK and PKC were unfortunately not confirmed in early clinical studies. Indeed, a general survey of all clinical trials applying new targeted and immune therapy to UM displayed disappointing results. This paper summarizes the most recent studies of UM-targeted therapies, analyzing the possible origins of failures. We also focus on hyperexpressed molecules involved in UM aggressiveness as potential new targets for therapy.

## 1. Introduction

Although overall survival (OS) of Uveal Melanoma (UM) patients is relatively high, reaching about 80% at 5 years, with high-risk UM patients frequently developing a metastatic disease, which has a severe prognosis. In about 90% of cases, metastases involve the liver, and untreated patients have a mean survival time of about 2 months, reaching about 6 months in treated patients [1,2,3]. While in metastatic cutaneous melanoma targeted therapies with B-Raf Proto-Oncogene, Serine/Threonine Kinase (B-RAF) and Mitogen-Activated Protein Kinase Kinase (MEK) inhibitors or immunotherapy with anti-Cytotoxic T-Lymphocyte Associated Protein (CTLA)-4 and/or anti- Programmed Cell Death (PD)-1/ Programmed Cell Death 1 Ligand (PDL)-1, treatments are highly effective [4], no effective standard treatment is available for metastatic UM so far. Indeed, a recent meta-analysis of OS after different treatments for metastatic UM showed no significant difference by treatment modality [5].

Recent advances in the understanding of the genetic differences between cutaneous and uveal melanoma point out the need of specific treatments for UM. In particular, mutually exclusive activating mutations involve the *GNAQ* or *GNA11* genes [6,7], which encode for Gα subunits of G-proteins and drive oncogenesis in most of UM. Additional driver mutations involve the *PLCB4* [8] or the Cysteinyl Leukotriene Receptor 2 (*CYSLTR2)* genes [9], in a minor fraction of UM cases.

In addition to driver mutations, monosomy of chromosome 3 [10,11,12], loss of chromosome 3 heterozygosity [13], and inactivating mutations of the BRCA1-associated protein 1 (*BAP1*) oncosuppressor gene [14] are highly associated with the metastatic risk. Differently, somatic mutations in *EIF1AX* and *SF3B1* genes specifically occur in UMs with disomy 3, which rarely undergo metastatic progression [15]. Importantly, *BAP1* loss-of-function mutations correlate with a distinct DNA methylation profile [16]. 

An important difference between uveal and cutaneous melanoma is related to the mutational load, which is typically high in cutaneous melanoma, in relation to UV exposure [17], and low in UM [16]. A high mutational load may result in the frequent generation of neo-antigens, which render the cutaneous melanoma highly immunogenic and sensitive to immune-checkpoint blockers such as anti-CTLA-4 [18,19] and anti-PD-1 monoclonal antibodies [20,21]. On the other hand, these immunotherapies have shown a low impact in metastatic UM outcome so far [22,23].

**Figure 1:**
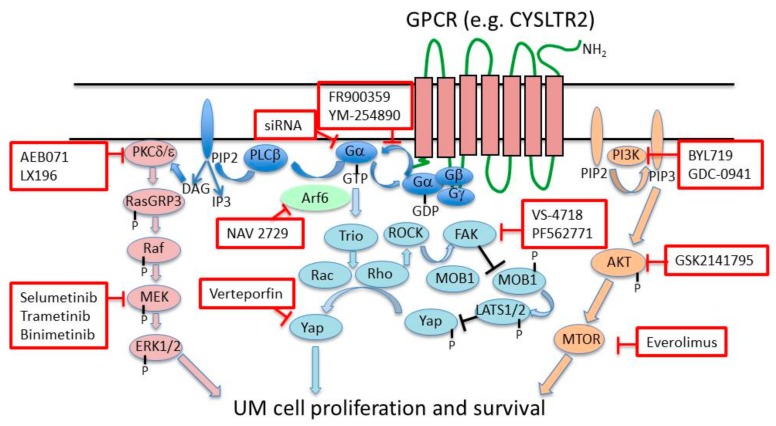
Main signaling pathways downstream GαQ or Gα11 and their inhibitors. Inhibitors of specific signaling molecules are depicted in red line boxes. GPCR: G protein-coupled receptor; CYSLT2R: Cysteinyl leukotriene receptor 2; PKCδ/ε: Protein kinase C delta/epsilon; RASGRP3: RAS guanyl releasing protein 3; PLCβ: Phospholipase C beta; DAG: Diacylglycerol; PIP2: Phosphatidylinositol biphosphate; IP3: Inositol 1,4,5-trisphosphate; ARF6: ADP ribosylation factor 6; TRIO: Trio rho guanine nucleotide exchange factor; RHO: Ras homologue family member; ROCK: Rho-associated, coiled-coil-containing protein kinase; Rac: Rac family small GTPase 1; FAK: Focal adhesion kinase; MOB1: MOB kinase activator 1; LATS: Large tumor suppressor kinase; Yap: Yes associated protein 1; PI3K: Phosphatidylinositol-4,5-bisphosphate 3-kinase. This figure was adapted from Yang et al. [1].

This review summarizes the status of targeted therapies, which are undergoing clinical testing in metastatic UM, and discusses new therapeutic possibilities emerging from recent advances in UM genetics and biology [3,24]. In particular, we discuss the possibilities to target oncogenic Gα proteins, Gα down-stream pathways, UM cell chromatin structure and transcriptional programs or overexpressed molecules involved in UM metastatic progression (Figure 1).

## 2. Targeting Driver Mutations

Targeting of driver mutations such as the *B-RAF V600E* by small-molecule inhibitors has provided a therapeutic option for a subset of cutaneous melanomas bearing such a mutation, although responses are transient. However, *B-RAF* mutations only rarely occur in UMs, which in about 90% of cases bear an activating mutation of the *GNAQ/GNA11* genes, driving tumor initiation [6,7]. In the GαQ or Gα11 Q209L mutant proteins, the catalytic glutamine is substituted by leucine, leading to the loss of Guanosine Triphosphate hydrolase (GTPase) activity. Therefore, these mutated proteins retain prolonged binding with GTP, leading to constitutive activation. The R183C mutation is less frequent and has been predicted to display a less strong inhibitory activity on GαQ or Gα11 [7]. *GNAQ/11* Q209L mutations are early or initiating events, which are present at any stage of UM [25,26]. However, *GNA11* mutations are more frequently found in UM metastases (57%) than *GNAQ* mutations (22%), suggesting that *GNA11* mutation is associated with higher metastatic risk [7]. In addition, Q209L mutations in *GNAQ* or *GNA11* have been found in 55% or 7% of blue nevi, respectively [6,7]. Mutated Gα proteins mediate the activation of the PLCα/PKC pathway and multiple downstream signaling pathways, including the RAF/MEK/ERK, PI3K/AKT/MTOR, and Trio/Rho/Rac/YAP1 pathways [3,27]. Therefore, mutated Gα proteins or downstream signaling molecules represent potential targets for therapy (Figure 1).

Less frequently, driver mutations involve the genes encoding for phospholipase C4 (*PLCB4*) [8] or the G protein-coupled receptor (GPCR) cysteinyl leukotriene receptor-2 (*CYSLTR2*) [9]. The identification of these alternative driver mutations provides further evidence for the pivotal role of the GPCR pathway in UM pathogenesis and could lead to new therapeutic possibilities in a fraction of UM [28]. The *PLCB4 D630Y* gain-of-function mutation is mutually exclusive with *GNA11* and *GNAQ* mutations, indicating PLCB4 as a downstream target of Gα proteins [8].

In view of their frequency as drivers in UM, *GNAQ/11* mutations may represent optimal targets for UM molecular therapies. However, the development of targeted therapy for mutated Gα proteins is still in an initial phase. The downregulation of *GNAQ* mutant expression using specific short interfering RNA (siRNA) decreased GαQ protein levels in UM cell lines, resulting in a decrease in Extracellular signal–Regulated Kinases (ERK) and AKT Serine/Threonine Kinase (AKT) signaling and in 5’ Adenosine Monophosphate-activated Protein Kinase (AMPK)-dependent autophagic cell death [29]. Other studies showed that the delivery of siRNA targeting mutated *GNAQ* through oncolytic viruses [30] or functionalized gold nanoparticles [31] inhibits UM cell viability and growth and may be useful for future gene regulatory therapeutic approaches.

The study of the GαQ-Q209L mutant showed a profound structural similarity between mutated and wild-type proteins, suggesting a limited possibility to generate specific inhibitors for the mutated form. The GαQ bearing the Q209P mutation is also GTPase-deficient and constitutively active but has distinct molecular characteristics. In particular, the different conformation in the switch II region of Gα-Q209P modifies the interaction with Gβγ and regulators of G-protein signaling, suggesting the possibility to develop specific targeting strategies for this mutant [32]. 

The cyclic depsipeptide FR900359, isolated from the *Ardisia crenata Sims* plant, specifically inhibits the activity of the wild-type GαQ, resulting in vasorelaxant effects on rat aortic arteries [33]. FR900359 inhibits GαQ/11/14 but not other mammalian Gα isoforms, by binding with high affinity to these molecules and acting as a pseudo-irreversible inhibitor. Importantly, it also inhibits ERK1/2 activation, cell proliferation, and migration and induces a more differentiated phenotype in melanoma cell lines with elevated Gα activity due to hyperexpression of a wild-type Gα or to Gα11-R183C or GαQ-Q209L mutations [34]. A more recent study found that FR900359 also suppressed signaling of mutated GαQ in UM cells, through the allosteric inhibition of the Guanosine Diphosphate/Guanosine triphosphate (GDP/GTP) exchange. In GαQ-mutated UM cells, FR900359 inhibited downstream signaling and cell proliferation and induced melanocytic differentiation. The differentiating activity involved the reactivation of polycomb repressive complex 2, which mediates gene silencing [35]. Similarly, another report showed that FR900359 inhibits oncogenic signaling in UM cells bearing activating mutations in either GαQ or Gα11 and induces cell cycle arrest and apoptosis. In addition, FR900359 prevented colony formation in a 3D-cell culture model of UM cells [36]. 

A very recent study reported that FR900359 predominantly blocks the ERK signaling pathway rather than the PLCβ pathway [37]. Importantly, FR900359 inhibited the growth of UM xenografts bearing the GαQ-activating mutation, leaving unaffected the growth of xenografts driven by mutated B-RafV600E. Altogether, these studies indicate that FR900359 or its derivatives may represent a new, potential treatment option for UM bearing activating *GNAQ* or *GNA11* gene mutations. The depsipeptide YM-254890 is structurally similar to FR900359 and is a selective inhibitor of the GαQ/11 G proteins. A recent study of the structure–activity relationship allowed the development of a simplified analogue YM-19, which proved the most effective GαQ/11 inhibitor among new analogues, opening new possibilities for the design of potent small-molecules suitable for pharmaceutical use [38].

## 3. Inhibition of Signaling Pathways Downstream Activated GαQ/11

Several signaling pathways are activated downstream of mutation-activated GαQ and Gα11, including the Mitogen-Activated Protein Kinase (MAPK), RAF/MEK/ERK, Phosphatidylinositol 3-Kinase/AKT/Mechanistic Target Of Rapamycin Kinase (PI3K/AKT/MTOR), and Trio Rho Guanine Nucleotide Exchange Factor/Ras Homolog Family Member/Rac Family Small GTPase 1/ Yes Associated Protein (Trio/Rho/RAC/YAP1) pathways, and provide several potential targets for therapy (Figure 1, Table 1).

The small GTPase and ADP-ribosylation factor 6, ARF6, is required for oncogenic GαQ signaling through all of these downstream pathways and also for β-catenin signaling. ARF6 mediates the trafficking of GαQ from the cell membrane to intracellular vesicles and of β-catenin to the nucleus. The ARF6-specific small molecule inhibitor NAV-2729 reduced UM cell proliferation in vitro and tumor growth in an orthotopic xenograft model. Therefore, ARF6 should be considered as a potentially actionable target for UM therapy [44].

Early preclinical studies demonstrated that MEK inhibitors such as selumetinib (AZD6244, ARRY-142886) [45] or TAK-733 [46] inhibit UM cell proliferation and viability in vitro, leading to the development of clinical studies of MEK inhibitors in metastatic UM. An initial clinical study of selumetinib in comparison to chemotherapy (NCT01143402) showed an improvement of response rate (RR) (14 vs. 0%) and of progression-free survival (PFS) (15.9 vs. 7 weeks), but limited effect on OS (11.8 vs. 9.1 months), in the selumetinib arm [39]. Another phase 1 trial, testing the effects of intermittent selumetinib, is now recruiting metastatic UM patients. The aim of this study is to test higher drug doses to more efficiently block the MAPK pathway and prevent the development of resistance (NCT02768766). A phase 1 dose-escalation study of the MEK inhibitor TAK-733 on 51 patients with advanced solid tumors, including 12 patients with UM, showed limited antitumor activity [47].

Further studies tested combinations of MEK inhibitors with other drugs. In the phase 3 study SUMIT (NCT01974752), 129 metastatic UM patients were randomized to receive selumetinib or placebo in combination with the alkylating drug dacarbazine. Selumetinib plus dacarbazine showed no significant improvement in the primary endpoint (PFS) compared with placebo plus dacarbazine [40]. According to a recent review of 590 case records from six eligible clinical studies, UM is poorly responsive to MEK inhibition independent of the type of inhibitor (selumetinib, trametinib, and binimetinib) and the drug combination used [48]. 

Preclinical models indicated a cooperative effect of selumetinib and paclitaxel in inducing tumor cell apoptosis [49]. On this basis, the SelPac clinical phase 2 study will investigate the activity of selumetinib in combination with paclitaxel in UM (EudraCT 2014-004437-22). The potential of new drug combinations to increase the efficacy of selumetinib was recently tested in patient-derived xenograft (PDX) models. Combinations of selumetinib with the ERK inhibitor AZ6197 and the mTORC1/2 inhibitor, vistusertib (AZD2014), were the most effective combination therapies [50].

Constitutively active Gα proteins trigger the Protein Kinase C (PKC) pathway, through diacylglycerol produced by PLCβ activation (Figure 1). Recent findings suggested that the PKCδ isoform has a predominant role in UM. Intriguingly, however, the PKCδ inhibitor B106 induced apoptosis in several UM cell lines, but apparently independent of activated PKCδ [51]. The pan-PKC inhibitor sotrastaurin (AEB071) decreased the viability of *GNAQ/GNA11-*mutated UM cells in preclinical studies [52]. A phase 1 study of AEB071 showed one partial response, 47% disease stabilizations, and a PFS of 15.4 weeks, in a cohort of 118 UM patients [53]. A further phase 1 study of the new-generation PKC inhibitor LXS196 is still recruiting patients (NCT02601378).

Although the PKC inhibitors AEB071 or AHT956 inhibited MAPK signaling and induced G1 arrest in *GNAQ/11*-mutated UM cells, they failed to induce UM tumor regressions in xenograft models. Instead, combinations of PKC and MEK inhibitors resulted in synergistic effects leading to tumor shrinkage in an UM model in vivo and provided the rationale for the use of these combinations in UM therapy [54]. However, a phase 1b/2 Study of AEB071 and binimetinib (MEK162) in metastatic UM was terminated before the initiation of the phase 2 part (NCT01801358).

The PI3K/AKT/MTOR pathway is constitutively activated by oncogenic Gα signaling. A preclinical study showed that the combination of the MEK inhibitor GSK1120212 and the pan-PI3K inhibitor GSK2126458 cooperatively induced apoptosis in the majority of UM cell lines, in a *GNAQ/11* mutant-dependent manner, while PI3K inhibition “per se” had limited effect [55]. Further, the PI3K-α inhibitor BYL719 blocked phosphorylation of AKT but had a limited antiproliferative activity in a panel of UM cell lines. However, its combination with the PKC inhibitor AEB071 showed synergistic effects on proliferation and survival of UM cells and inhibited tumor growth in a *GNAQ*-mutated UM xenograft model [56]. These findings supported the requirement of a simultaneous inhibition of PI3K and different downstream pathways for combination therapies of UM and promoted the design of a clinical phase 1 study to define the safety, tolerability, and maximum tolerated dose (MTD) of the AEB071 and BYL719 drug combination in metastatic UM (NCT02273219).

The combination of selumetinib with the AKT inhibitor MK2206 induced activation of AMP-activated protein kinase and resulted in the synergistic induction of autophagic cell death in UM cells in vitro [29]. In addition, this combination was effective in inhibiting UM growth in xenograft models. These data led to the design of a phase 2 clinical trial of trametinib with or without the AKT inhibitor GSK795, but this combination failed to improve PFS and RR with respect to the single selumetinib arm [41 (NCT01979523).

Other studies focused on MTOR as potential target for combination therapies. A clinical phase 2 study (NCT01252251) tested whether combined treatment with the MTOR inhibitor everolimus and the somatostatin receptor agonist pasireotide would be efficacious in metastatic UM. This combination had limited clinical benefit in 13 evaluable patients and required dose reductions for side-effects [42]. A combination screening with several small molecule inhibitors inhibiting PKC, MEK, AKT, PI3K, and MTOR in a panel of UM cell lines showed a potent synergy between the MTOR inhibitor everolimus and the PI3K inhibitor GDC0941. This drug combination showed enhanced pro-apoptotic effects in vitro and in two PDX models. The molecular basis of this synergy was related to the ability of GDC0491 to block the reactivation of AKT induced by everolimus [57]. Another screening study using a panel of UM PDXs found that the two small molecules CGM097 (p53-MDM2 inhibitor) and RAD001 (mTORC1 inhibitor) has synergistic effects with the PKC inhibitor AEB071, demonstrating tumor regression in UM PDXs [58]. These studies suggest new potential combinational therapies for metastatic UM.

The Hippo oncosuppressor pathway limits organ size through a kinase cascade involving serine/threonine kinase 4 (MST1/2) and Large Tumor Suppressor Kinase 1/2 (LATS1/2 kinases) [59]. The LATS1/2 kinases inactivate by phosphorylating YAP and Transcriptional Co-Activator With PDZ-Binding Motif (TAZ), which have oncogenic potential related to their ability to co-activate gene transcription. Indeed, high YAP/TAZ expression and nuclear localization has been found in several tumor types [60,61,62,63], including UM [64,65]. Mutation-activated GαQ or Gα11 induced YAP/TAZ dephosphorylation and signaling, which is an essential mediator of oncogenic activity in UM development. The activation of YAP by activated GαQ involves a Trio-Rho/Rac signaling circuit, which stimulates actin polymerization independently upon the activation of PLCβ and the Hippo pathway [65]. Knockdown of mutated GαQ decreased the nuclear localization of YAP and its interaction with the transcription factor TEA domain transcription factor (TEAD) [65], which is essential for YAP-mediated proliferation, epithelial–mesenchymal transition, and oncogenesis [66]. Moreover, the YAP inhibitor verteporfin, inhibited the growth and tumorigenesis of GαQ/11-mutated UM cells [64,65,67]. Altogether, these data indicate that YAP is a potentially useful target for the therapy of UM-bearing mutations in *GNAQ* or *GNA11* genes [68,69].

A recent study showed that GαQ activates the focal adhesion kinase Focal adhesion kinase (FAK), whose activity is essential for YAP activation and UM cell growth. Ablation of the FAK-encoding gene (*PTK2*) or blockade of FAK activity by the small molecules VS-4718 or PF562771 inhibits YAP signaling and UM growth [70], suggesting that FAK is a new actionable target in UM and for other GαQ-dependent diseases.

## 4. Targeting Chromatin Structure and Transcription

The transcriptional programs that mediate tumor progression require changes in the expression or function of transcription factors and several chromatin structure regulators [71]. In UM, which typically has a low mutational burden [16], epigenetics alterations, including changes in the DNA methylation or histone acetylation status, may play a relevant pathogenic role [72,73]. Therefore, the targeting of transcriptional regulators may represent an attractive therapeutic option in UM.

Histone deacetylases (HDAC) are a family of enzymes that remove acetyl groups from acetylated lysine residues of histone proteins and act as epigenetic regulators. In general, deacetylated histones can bind to the DNA more tightly, thus limiting the access of transcription factors and repressing gene transcription. On the other hand, histone acetylation is associated with gene transcription. Human HDACs are divided into four classes, displaying different sensitivity to synthetic inhibitors, and play a relevant role as epigenetic modifiers in cancer [74]. In UM, several HDAC inhibitors, including valproic acid, panobinostat, vorinostat [75], tricostatin A [75,76], tenovin-6 [77], depsipeptide [78], MS-275 [79], quisinostat [80], JSL-1 [81], MC1568, and MCI1575 [82], have shown antitumor activities, including growth arrest or apoptosis in vitro and/or suppression of UM xenograft growth [83]. Interestingly, some of these HDAC inhibitors induce a shift of the gene expression profile from a high-risk (class-2) to a low-risk (class-1) profile in primary UM cell cultures [75]. A recent report showed additive cytotoxic effects by combining neratinib (an inhibitor of Her2 and Epidermal Growth Factor Receptor (EGFR) tyrosine kinases) with the HDAC inhibitor entinostat in PDX of UM. This drug combination cooperatively induced the internalization and degradation of Gα proteins and EGFR and also targeted the Ras pathway, thereby activating mitochondrial dysfunction and autophagy [84]. EGFR is frequently overexpressed in UM and has been proposed as a potential target [85].

Based on the encouraging results of preclinical studies, clinical studies with HDAC inhibitors are ongoing. A phase 2 study of oral vorinostat in metastatic UM had the primary objective to determine the overall RR. It enrolled 23 patients and was terminated in 2018, but the results are not yet available (NCT01587352). A proof of concept study of vorinostat in patients with class 2 high-risk UM will assess the possible switch from a class 2 gene expression profile into a profile that resembles normal melanocytes on fine needle aspirate biopsies (NCT03022565) as observed for cell lines treated with HDAC inhibitors [75]. A randomized phase 2 trial will assess the effects of adjuvant sunitinib or valproic acid in preventing high-risk UM from metastatic spreading by evaluating OS at 2 years (NCT02068586). A multicenter phase 2 open label study has been planned to evaluate efficacy of the concomitant use of the anti-PD1 pembrolizumab and entinostat in patients with metastatic UM (NCT02697630). The rationale for this study relies not only on the direct effect of HDAC inhibitors on UM cells, but also on their immune-modulatory activity, which may cooperate with the anti-PD-1 effects [86]. 

Alterations of DNA methylation represent another important epigenetic mechanism of cancer development [87]. In general, gene promoter hypermethylation may induce silencing of oncosuppressor genes. These mechanisms can be a target for epigenetic therapy with drugs such as azacitidine and its derivatives that inhibit DNA methyltransferase I and mediate hypomethylation of DNA. On the other hand, hypomethylation has also been implicated in the development and progression of cancer through the activation of oncogene expression. Examples of genes silenced by hypermethylation in UM are *INK4a* [88,89], *TIMP3* [90], and *S100A2* [91]. The re-expression of these genes can be induced by in vitro treatment with azacitidine or its derivatives. In addition, treatment with demethylating agents may reduce the growth and invasiveness [92] or enhance the sensitivity of UM cells to the cytotoxic activity of IFN-γ in vitro [91].

A recent study using a multiplatform analysis of 80 primary UM found that *BAP1* loss follows monosomy 3 in poor-prognosis cases and correlates with a peculiar global DNA methylation profile. Instead, a better-prognosis monosomy 3 cohort bearing *EIF1AX*- and *SRSF2/SF3B1*-mutant UM showed a distinct DNA methylation profile [16]. Another study performed on 87 UM samples revealed phylogenetic clusters of global DNA methylation that correlated with the gene expression profile (class-1 versus class-2) and with *BAP1*, *SF3B1* or *EIF1AX* mutations [93]. Altogether, these studies indicate that the global methylation profile is related to the almost mutually exclusive mutations in *BAP1*, *SF3B1* or *EIF1AX* genes, which are associated with a distinct metastatic risk. Therefore, the DNA methylation status may be important to modulate the metastatic behavior in UM, and drugs altering the DNA methylation profile may deserve further studies in high-risk UM, particularly in an adjuvant setting.

BRCA1 associated protein 1 (BAP1) has a histone deubiquitinase activity, which results in tumor and metastasis suppressor activity. A recent study used the transposase-directed transposon insertion to map the BAP1 target genes in UM. This “calling card technique” allowed generating a list of BAP1 genomic targets that provides new insights into pathways leading to UM metastasis and novel potential therapeutic targets [94]. For example, the genes involved in the epithelial-to-mesenchymal transition signature were upregulated.

Among regulators of gene transcription, the bromodomain and extraterminal (BET) protein family members bromodomain containing 2, 3, 4 (BRD2, BRD3, BRD4) and Bromodomain Testis Associated (BRDT) promote transcriptional elongation by binding to acetylated lysine of histones in chromatin and recruiting transcriptional protein complexes. Pharmacological inhibitors of these proteins suppress the growth of different tumors and may represent potential anticancer drugs [95,96]. 

In particular, the BRD4 inhibitor JQ1 [97] has shown cytotoxic activity in UM cells bearing *GNAQ/11* mutations, while it had limited effects in unmutated cells. JQ1 inhibits the expression of genes involved in the regulation of cell cycle, apoptosis, and DNA repair, such as *BCL-XL* and *RAD51.* JQ1 was also effective in mouse xenograft models of *GNAQ*-mutant UM, supporting the concept that BRD4 targeting is a novel therapeutic possibility [98].

As JQ1 has a short half-life and was not suitable for clinical trials, the second-generation BET inhibitor PLX51107 [99] is now undergoing clinical testing in patients with different advanced cancers, including UM patients (NCT02683395). So far, however, patients with liver metastases of UM showed resistance to BET inhibitors and progressed following treatment. A very recent report addressed the mechanisms of metastasis resistance to BET inhibitors by focusing on soluble factors in the liver microenvironment [100]. Fibroblast growth factor 2 (FGF2), which is produced by hepatic stellate cells, reduced the responsiveness of UM cells to the antiproliferative effect of BET inhibitors. In addition, treatment with BET inhibitors increased expression of the FGFR pathway in vitro and in UM patients. The combination of the FGFR inhibitor AZD4547 with PLX51107 inhibited the growth of UM cells co-implanted with human stellate cells in immune-deficient mice. These data provide the basis for new combinational treatments of UM liver metastases through the concomitant inhibition of BRD4 and FGFR pathways [100].

A study of kinome expression profiling in JQ1-treated UM cells identified a repressed kinase network, comprising several cell cycle-regulated protein kinases. Accordingly, UM cells were sensitive to inhibitors of these protein kinases, including the PLK1 inhibitor BI6727 (volasertib). Therefore PLK1, and possibly other mitotic kinases, may represent new potential drug targets in UM [101].

## 5. Targeting Hyper-Expressed Molecules Involved in Progression

Several studies of gene or protein expression led to the identification of molecules that are overexpressed in primary UM, which progressed to a metastatic disease. Some of these molecules have been functionally linked to pro-invasive properties of UM cells. For example, the analysis of the gene expression profiling of primary UM showed higher expression of the *SDCBP* gene (encoding for mda-9/syntenin). Gene silencing showed an important role of mda-9/syntenin in UM cell migration, invasion, and FAK activation, suggesting that mda-9/syntenin is involved in uveal melanoma progression and may represent a potential therapeutic target [102]. Mda9/syntenin was identified as a prometastatic molecule in cutaneous melanoma [103] and then in different tumors [104]. It is a multidomain adaptor protein, which binds to different oncogenic signaling molecules through its PDZ1 and PDZ2 domains and mediates a pro-invasive behavior [105]. Recent studies showed that peptide [106] or small-molecule [107] PDZ inhibitors block mda-9/syntenin functions and the invasiveness of different cancer cells, although they have not yet been tested in UM models.

Several matrix metalloproteinases (MMPs) are involved in invasiveness, angiogenesis, and progression of different tumors, including UM, through the degradation of extracellular matrix components [108]. Early studies showed that MMP-2 [109] and/or MMP-9 [110,111] are expressed in primary UM, and high expressions correlate with a dismal prognosis. Further experimental evidence indicated that MMPs mediate UM cell invasiveness in vitro [112] and in xenograft models. Indeed, inhibition of MMP2 expression and/or activity by epigallocatechin gallate [113] or zeaxanthin [114] inhibited UM cell invasiveness, supporting a potential role of MMP2 inhibitors in the prevention of UM metastases. 

Membrane localization of MMP14 is induced by protein tyrosine phosphatase 4A3 (PTP4A3). Inhibition of MMP14 expression decreased UM cell invasiveness, suggesting that PTP4A3-mediated localization of MMP14 is relevant for metastasis induction [115]. 

The expression of a disintegrin and metalloproteinase *(ADAM)10* gene [116] or protein [117] in primary UM correlated with metastasis development and a poor survival. In addition, *ADAM10* silencing inhibits UM cell invasion in vitro, indicating that ADAM10 may contribute to UM progression and may represent a potential target for adjuvant therapy [116]. 

Notwithstanding our increasing knowledge on the role of metalloproteinases in tumor progression, the enthusiasm for MMP/ADAM inhibitors as anticancer agents has been weakened by the results of early clinical trials of broad-spectrum MMPs inhibitors. Novel drug-design strategies may allow the development of more specific MMPs or ADAM inhibitors suitable for future clinical studies [118]. In this regard, MMP/ADAM inhibitors should be preferably tested in an adjuvant setting.

The tyrosine kinase receptor c-Met and its ligand hepatocyte growth factor (HGF) mediate proliferation, survival, and pro-invasive activity of several tumors [119] including UM [120]. *MET* mRNA [116] or protein [121,122,123] expression in primary UM correlates with a higher incidence of metastases, and UM liver metastases strongly express c-Met [124]. In addition, soluble c-Met ectodomain increased in the sera of UM patients with liver metastases relative to patients without metastases, thus representing a potential biomarker of progression [125]. Crizotinib, an inhibitor of c-Met, Anaplastic Lymphoma Receptor Tyrosine Kinase (ALK), and ROS Proto-Oncogene 1, Receptor Tyrosine Kinase (ROS1), inhibited the phosphorylation of c-Met, suppressed in vitro migration of UM cells, and reduced metastasis formation in UM xenograft models [126]. These data suggested crizotinib as an adjuvant therapy for UM patients at high risk of metastases. Therefore, a phase 2 clinical trial of crizotinib has been designed but is not yet recruiting, in high-risk UM following therapy of the primary tumor (NCT02223819).

The IGF-1R has been also implicated in UM progression, as IGF1-R is expressed in UM metastases. Both endogenously produced or exogenous IGF-1-mediated AKT phosphorylation and proliferation of a UM cell line derived from a metastasis and an anti-IGF-1R blocking mAb inhibited these effects [127]. Moreover, two independent studies showed that the cyclolignan picropodophyllin, an inhibitor of IGF-1R, suppresses the growth of UM cells in vitro and in xenograft models [128,129]. Altogether, these studies support the potential benefit of IGF-1 or IGF-1R blockade in UM therapy.

Anti-angiogenic therapy is currently used for the treatment of metastases of several malignancies, in combination with other therapies. Angiogenesis may represent a potential target for therapy also in UM [130], which is highly vascularized and expresses angiogenesis-related genes [131]. Early observations reported increased concentrations of Vascular Endothelial Growth Factor A VEGF-A in aqueous humor in UM patients [132]. In addition, VEGF-A is expressed by UM cells and is upregulated by hypoxia via HIF-1alpha [133]. VEGF-A serum levels were higher in UM patients with metastases than in nonmetastatic ones, suggesting that VEGF-A could be involved in metastasis development [133,134]. Indeed, the anti-VEGF-A-blocking mAb bevacizumab inhibited in vitro invasion of UM cells and suppressed the formation of liver micrometastases in an experimental model in vivo [135].

Local therapy with anti-angiogenic agents is broadly used for treating or preventing the complications of radiation therapy of primary UM. However, intravitreal bevacizumab in large primary UMs showed paradoxical tumor growth [136]. In addition, in a preclinical model mimicking UM, intraocular injection of bevacizumab paradoxically stimulated melanoma growth in murine eyes, possibly due to the upregulation of pro-angiogenic factors [137]. Altogether, these data suggest caution in the use of intravitreous bevacizumab for UM in the neoadjuvant setting.

Two studies of anti-angiogenic drugs addressed the treatment of metastatic UM. Aflibercept is a soluble VEGF receptor, which can trap VEGF and inhibit angiogenesis. A multicenter phase 2 study of aflibercept in patients with inoperable stage III or IV metastatic melanoma included ten patients with UM. This study reported a PFS of above 4 months in 50% of the UM cohort, suggesting potential activity [138]. A phase 2 Trial of bevacizumab in combination with temozolomide as first-line treatment in patients with metastatic UM failed to reach the primary endpoint of 6-month PFR of 40% [139]. 

The overall modest effects of anti-VEGF/VEGFR therapies in metastatic UM may be explained by the activation of alternative pro-angiogenic pathways. A preclinical study showed that not only VEGF but also angiopoietin protein like-4 (ANGPTL4) was highly induced by hypoxia in UM cells and was present in vitreous samples from UM patients [140]. Inhibition of either VEGF or ANGPTL4 alone reduced UM cell-driven angiogenesis in vitro, but the inhibition of both angiogenic factors showed additive effects. Therefore, co-targeting of both VEGF and ANGPTL4 may be required to block angiogenesis in UM. Moreover, the UM microenvironment can use IL-8 as an additional pro-angiogenic pathway [141]. Signaling through HGF/MET may also cause resistance to anti-VEGF therapies [142], and the simultaneous inhibition of VEGFR and MET results in enhanced efficacy [143]. Cabozantinib (XL184; Exelixis, Inc.) is a multi-tyrosine kinase inhibitor that co-targets MET, AXL Receptor Tyrosine Kinase (AXL), and VEGFRs. In a phase 2 randomized discontinuation trial of cabozantinib in metastatic melanoma, 13 of the 22 evaluable patients (59%) with UM showed a reduction of at least one target lesion. The median OS for the 23 patients with UM enrolled was 12.6 months, and the median PFS was 4.8 months with an overall disease control rate of 61%, suggestive of a potential activity of cabozantinib in UM [143]. A phase 2 randomized study of cabozantinib compared with temozolomide or dacarbazine in patients with metastatic UM has been completed, but results are not yet available (NCT01835145).

Another multikinase inhibitor, sunitinib, targets platelet-derived growth factor (PDGF)Rs, VEGFRs, and CD117 (c-KIT), which are expressed in the majority of metastatic UM. In a pilot trial, twenty patients with metastatic UM expressing c-KIT received daily sunitinib malate in 4-week cycles. There was one partial response and 12 stable diseases (SD), and the median OS and PFS were of 8.2 and 4.2 months, respectively. Intriguingly, no correlation between c-KIT expression and clinical outcome was found, suggesting the possible importance of other TK targets [144]. Another study of adjuvant sunitinib compared OS in high-risk UM patients with a retrospective cohort of institutional controls with the same risk factors. In this trial, adjuvant sunitinib was associated with a better OS [145]. 

The multikinase inhibitor sorafenib, which targets VEGFR, PDGFR, and Raf kinases, was also tested in advanced UM. However, a clinical phase 2 study showed only disease stabilizations, significant toxicity, and no improvement of the quality of life [146]. Further, the combination of sorafenib with carboplatin and paclitaxel (NCT00329641), showed only minor responses and stabilizations, without impact on survival [43]. The ubiquitin-like protein NEDD8 (neural-precursor cell-expressed developmentally downregulated 8) is conjugated to different target proteins through a process called neddylation, by NEDD8 Activating Enzyme E1 Subunit 1, 2 (NAE1 and NAE2) enzymes. This process is similar to ubiquitination and has been involved in tumor progression and angiogenesis [147]. A recent report showed that NAE1 is highly expressed in UM cells, and that blockade of neddylation by the small molecule MLN4924 inhibited the stem-cell phenotype, paracrine secretion of VEGF-C, and angiogenic properties in UM cells. Inhibition of neddylation suppressed the development of hepatic metastases in an UM xenograft model, suggesting that the neddylation pathway represents a new target for the prevention or treatment of metastasis in UM [148].

Several lines of evidence indicated a possible role for the chemokine receptors CXCR4 and CCR7 in the metastatic progression of UM. CXCR4 protein expression was detected in a fraction of UM primary tumors, in relation to an epithelioid-mixed UM cell phenotype [149]. In vitro studies showed that hypoxia increased migration and invasiveness of UM cells through the HIF-1α-dependent expression of chemokine receptor CXCR4 [150]. An arylsulfonamide compound (64B) inhibits the hypoxia-inducible expression of *MET* and *CXCR4* and reduces primary UM growth and metastasis in xenograft models. The compound 64B interferes with HIF-1α binding to p300/CBP co-factors and inhibits p300 recruitment and activation of the *MET* and *CXCR4* gene promoters. A further optimization of the 64B scaffold may allow the development of new compounds for the UM treatment [151]. An antibody blocking the CXCR4 ligand CXCL12 or siRNA targeting *CXCR4* expression [152] inhibited migration of UM cells by liver extracts. In addition, a small molecule, partial antagonist of CXCR4, inhibited metastasis formation in different xenograft tumor models, including UM [153].

More recent data indicate that *CXCR4* expression is epigenetically regulated in the tumor microenvironment [154]. CCR7 expression in primary UM is associated with poor survival, suggesting that it may represent a target for therapy [155]. Although UM cell lines express CXCR4 and CCR7, which are relevant for hepatic metastases formation, factors present in the hepatic microenvironment reduced CXCR4 and CCR7 expression on UM cells [156].

## 6. Conclusions

Metastatic UM still remains an incurable disease, since no therapies have shown a significant impact on survival so far [1,2,3,5]. In particular, the failure of MEK inhibitors to significantly improve survival [45] may relate to the redundancy of signaling pathways downstream the mutated Gα proteins. Therefore, combinational therapies that co-target multiple pathways are currently under investigation. Recent preclinical studies provided new chances of directly blocking mutated Gα proteins activity and their downstream signaling pathways through the natural depsipeptide FR900359, which acts as an allosteric inhibitor of Gα [34]. However, FR900359 is active on both activation-mutated and nonmutated Gα proteins [33,34], raising the question about potential side-effects. Another potentially interesting actionable target is ARF6, which is necessary for Gα protein signaling through all downstream pathways [44].

High-risk UM-bearing *BAP1* mutation and chromosome 3 monosomy displayed distinct epigenetic [16] and gene expression profiles [157,158]. These findings indicate that modifiers of chromatin structure or gene transcription may represent useful tools to revert high-risk UM phenotype and inhibit aggressiveness. Preclinical studies using different HDAC [83] or BET inhibitors [98,100] showed promising results in UM preclinical models, and several clinical trials are already ongoing. 

Other potential targets are hyper-expressed molecules associated with UM metastatic behavior. Preclinical studies validated several of these molecules as potential targets for inhibiting UM invasiveness and metastatic progression. In several instances, however, preclinical UM models are hampered by the frequent usage of nonmonosomic cell lines, which are poorly representative of the high-risk UM. In this respect, the development of UM Patient Derived Xenograft (PDX) models, particularly from high-risk primary UM or from liver metastases [159], may provide better preclinical tools for testing new therapies.

The possibility to identify high-risk patients through the assessment of monosomy 3, *BAP1* status or gene expression profiling opens up the possibility of testing agents inhibiting UM invasiveness and progression in an adjuvant setting. Strategies for the prevention of metastasis must consider that at the time of diagnosis, eventually life-threatening micrometastases have already formed. Therefore, a treatment inhibiting invasiveness will not have major therapeutic effects unless it also inhibits the growth of these micrometastases. In this respect, the hepatic microenvironment may contribute to the resistance of UM metastases to different treatments, as it provides multiple growth and survival factors to UM cells, including HGF, FGF2, IGF-1, and CXCL12. A recent study reported that several inflammatory and profibrogenic mediators are important in the homing of UM cells to the liver and are part of a bidirectional crosstalk between UM cells and hepatic stellate cells [160]. It is hoped that a better knowledge of this metastatic microenvironment may provide new targets for UM therapy.

## Figures and Tables

**Table 1:** Examples of targeted therapy clinical trials in Uveal Melanoma (UM).

Clinical Trial	Intervention (N° of Patients)	Phase	Status [Reference]	Date
MEK inhibitors				
NCT01143402	Selumetinib in comparison to chemotherapy (120)	Phase 2	Completed, has results [39]	2010–2017
NCT02768766	Intermittent Selumetinib (28)	Phase 1	Recruiting	2016–2019
NCT01974752	SUMIT ^1^ selumetinib or placebo in combination with dacarbazine (152)	Phase 3	Completed, has results [40]	2013–2017
EudraCT 2014-004437-22	selumetinib and paclitaxel (72 planned)	Phase 2	Ongoing, no longer recruiting	2015–2019
PKC inhibitors				
NCT02601378	LX196 as a single agent and in combination with HDM201 ^2^ (122 estimated)	Phase 1	Recruiting	2015–2019
PI3K/AKT/MTOR inhibitors				
NCT01801358	AEB071 and Binimetinib, MEK162 (38)	Phase 1b/2	Terminated for scientific reasons before the initiation of the Phase II	2013–2016
NCT02273219	AEB071 and BYL719 (30 estimated)	Phase 1	Active, not recruiting	2014–2018
NCT01979523	Trametinib with or without GSK2141795 (44 estimated)	Phase 2	Completed [41]	2013–2018
NCT01252251	RAD001, Everolimus and Pasireotide, SOM230 LAR (14)	Phase 2	Completed, has results [42]	2010–2017
HDAC ^3^ inhibitors				
NCT01587352	Vorinostat, NSC 701852 (23)	Phase 2	Terminated	2012–2018
NCT03022565	Vorinostat (10 estimated)	Early phase 1	Recruiting	2019–2026
NCT02068586	Adjuvant Sunitinib or Valproic acid (150 estimated)	Phase 2	Recruiting	2014–2021
NCT02697630	Pembrolizumab and Entinostat (29 estimated)	Phase 2	Active, not recruiting	2018–2023
BET ^4^ inhibitors				
NCT02683395	PLX51107 (50)	Phase 1b/2a	Terminated (Business Decision)	2016–2018
Tyrosine kinase receptor MET ^5^				
NCT02223819	Crizotinib (34 estimated)	Phase 2	Active, not recruiting	2015–2019
Multi-tyrosine kinase inhibitors				
NCT01835145	Cabozantinib compared with Temozolomide or Dacarbazine (47)	Phase 2	Completed	2013–2016
NCT02517736	Sorafenib, Nexavar^®^ (32 estimated)	Phase 2	Completed	2012–2015
NCT00329641	BAY 43-9006, Sorafenib With Carboplatin and Paclitaxel (25)	Phase 2	Completed, has results [43]	2011–2012
EudraCT 2008-008794-55	Sunitinib versus Dacarbazine (84)	Phase 2	ended	2010–2014

^1^ Selumetinib in Metastatic Uveal Melanoma; ^2^ human double minute 2 homolog (HDM2) inhibitor; ^3^ Histone Deacetylase; ^4^ Bromodomain and Extra-Terminal motif; ^5^ MET Proto-Oncogene, Receptor Tyrosine Kinase.
